# 87842 The Effects of Head Impact Exposure on Changes in Neurocognitive and Oculomotor Function Across a High School American Football Season: A Pilot Study

**DOI:** 10.1017/cts.2021.687

**Published:** 2021-03-30

**Authors:** Megan E. Huibregtse, Jonathan T. Macy, Jesse A. Steinfeldt, Keisuke Kawata

**Affiliations:** School of Public Health--Bloomington, Indiana University

## Abstract

ABSTRACT IMPACT: This work reveals the influence of a season of American football-related head impact exposure on two functional outcome measures in a cohort of adolescent boys, shedding light on the chronic effects of 'subconcussive head impacts.' OBJECTIVES/GOALS: To examine the influence of a season of exposure to head impacts in American football on changes in neurocognitive and oculomotor function in adolescent male athletes. METHODS/STUDY POPULATION: Participants were recruited from a local high school: the football group (FB; n = 26) was instrumented with sensor-installed mouthguards to track impact exposure during games and practices, and members of the men’s cross-country team were recruited to the control group (CON; n = 9). All participants were administered Immediate Post-concussion Assessment and Cognitive Testing (ImPACT) and were assessed for near point of convergence (NPC) at pre- and post-season. Linear models will be fit for changes in the five ImPACT composite scores and NPC values, with group and one of the head impact variables as predictors for each model. In a secondary within-group analysis, correlation coefficients will be calculated for the relationships between the head impact variables and the functional change scores for the FB group. RESULTS/ANTICIPATED RESULTS: The two groups did not differ significantly on age or number of previous concussions; the CON group had significantly lower BMI. Group assignment was significantly associated with change in NPC (p < 0.05 for all three models); no significant associations were observed for any of the head impact variables with change in NPC. Group and each of the head impact variables (total impacts, sum of peak linear acceleration [PLA], and sum of peak rotational acceleration [PRA]) were not significantly associated with change in any of the five ImPACT composite scores. Change in visual memory composite score was negatively correlated with total impacts (r = -0.37, p = 0.034) and sum of PRA (r = -0.36, p = 0.040). DISCUSSION/SIGNIFICANCE OF FINDINGS: Significant, albeit relatively weak, correlations between change in visual memory composite score and two head impact kinematic variables, coupled with significant increases in NPC in the FB group compared to the CON group, suggest that a season of exposure to football-related head impacts has the potential to elicit minor functional impairments.

